# Modelling Inductive Sensors for Arc Fault Detection in Aviation

**DOI:** 10.3390/s24082639

**Published:** 2024-04-20

**Authors:** Gabriel Barroso-de-María, Guillermo Robles, Juan Manuel Martínez-Tarifa, Alexander Cuadrado

**Affiliations:** 1 Airbus Defence and Space, 28906 Getafe, Spain; gabriel.barroso@airbus.com; 2 Department of Electrical Engineering, University Carlos III of Madrid, 28911 Leganes, Spain; jmmtarif@ing.uc3m.es; 3 Escuela de Ciencias Experimentales y Tecnología, University Rey Juan Carlos, 28008 Madrid, Spain; alexander.cuadrado@urjc.es

**Keywords:** series arcs, inductive sensors, more-electric aircraft, all-electric aircraft, modelling, finite elements method

## Abstract

Modern aircraft are being equipped with high-voltage and direct current (HVDC) architectures to address the increase in electrical power. Unfortunately, the rise of voltage in low pressure environments brings about a problem with unexpected ionisation phenomena such as arcing. Series arcs in HVDC cannot be detected with conventional means, and finding methods to avoid the potentially catastrophic hazards of these events becomes critical to assure further development of more electric and all electric aviation. Inductive sensors are one of the most promising detectors in terms of sensitivity, cost, weight and adaptability to the circuit wiring in aircraft electric systems. In particular, the solutions based on the detection of the high-frequency (HF) pulses created by the arc have been found to be good candidates in practical applications. This paper proposes a method for designing series arc fault inductive sensors able to capture the aforementioned HF pulses. The methodology relies on modelling the parameters of the sensor based on the physics that intervenes in the HF pulses interaction with the sensor itself. To this end, a comparative analysis with different topologies is carried out. For every approach, the key parameters influencing the HF pulses detection are studied theoretically, modelled with a finite elements method and tested in the laboratory in terms of frequency response. The final validation tests were conducted using the prototypes in real cases of detection of DC series arcs.

## 1. Introduction

Modern aircraft design is already ruled by more-electric aircraft (MEA), which brings about a substantial increase in electrical power [1]. Additionally, the new aircraft concept based on hybrid and full electrical propulsion (all-electric aircraft, AEA) [2,3] is also being progressively consolidated. The main feature regarding the electrical design for MEA and AEA is the incorporation of high-voltage architectures as an answer to the huge increment of on-board power demand [4]. Since increasing the current is ruled out because of the consequent cable gauge increase, the working voltage of modern architectures has been raised to satisfy the new power requirements. Currently, high-voltage and direct current (HVDC) architectures are leading the trend, with levels of 270 VDC for non-propulsive purposes and up to 1–3 kVDC for electrical propulsion. These architectures provide a lot of benefits, namely enabling the integration of high-consuming loads, allowing the substitution of hydraulic and mechanic equipment by electrical equipment, and reducing the wiring gauge, which entails more capabilities for the aircraft to save weight and volume onboard [3,5]. Nevertheless, this technology is still facing some challenges, like the physical effects caused by the high energy management at extreme conditions (low pressure), which facilitates the apparition of ionisation phenomena such as partial discharges and arc faults. Precisely, Paschen’s Law states that at low pressures and high voltages it is easier to have a dielectric rupture and, therefore, electric arcs [6].

Arcs are formed when the voltage difference across the gap is high enough to exceed the dielectric strength of the gas, allowing the electric current to ionise the medium in the gap. This ionisation creates a plasma channel, whose high temperature can break down the wire’s insulation and possibly trigger an electrical fire [7], meaning a hazard for the aircraft integrity.

Arc faults are generally classified into two types: parallel and series [8,9]. A parallel arc is produced if the dielectric breakdown leads to a discharge between different conductors with different potentials, whereas a series arc happens through different parts of the same conductor, typically after an anomalous interruption of the circuit.

Common causes of arcing in aviation are ageing of electrical components, thermal effects such as thermal expansion, electrical faults, mechanical faults, abrasion of the conductive parts, vibrations, and contamination by aircraft fluids [10,11]. It has been found that cables are involved in about a third part of the arc events [10], and it is worth mentioning that whereas many aircraft parts are periodically inspected or renewed, due to its complexity, the wiring system remains usually untouched, thus favoring the conditions for the occurrence of electrical arc faults [12]. After all, the problems of wiring harnesses accounted for more than 30% of the total of aircraft failures [13,14], and fires or explosions resulting from arcing events in cable bundles are the suspected cause of several commercial and military aircraft incidents and accidents [10,15].

The arc fault phenomenon is already addressed for conventional power networks at 115 VAC and 28 VDC. In fact, parallel arcing can be current limited due to the available overcurrent protection elements like circuit breakers, and dedicated devices such as the Arc Fault Circuit Breaker (AFCB) [16].

Unfortunately, these devices are not fully applicable for modern architectures. On the one hand, AFCB does not protect for series arcs, and on the other hand, the series arcing currents do not reach the magnitude for tripping conventional thermal or magnetic circuit breakers [16]. Additionally, the MEA and AEA networks strongly enhances the probability of arc apparition since they are directly related to the magnitude of voltage in the wiring and other ambient factors like pressure or humidity [17]. Also, the severity of the arc faults is higher in DC, given the fact that operation in AC eases the self-extinction in the zero-crossing of the current. As a result, a series fault at high voltage can be very destructive, since it is hard to extinguish and liberates a large amount of energy [18]. Then, the combination of high-voltage architectures, DC topology, and environmental constraints make the new electrical networks within aircraft prone to arc fault problems.

According to the aforementioned reasons, it is critical for MEA and AEA to have the capability to detect and isolate electrical faults to meet the demanding safety standards required in aviation. The difficulty of the arc phenomena detection must be remarked upon, because of its erratic behaviour [19], and also the highly demanding requirements for aviation: high detection rate (>99%), fast response (hundreds of milliseconds), low false trips, no perturbing the network, and light and small detectors, which hamper new developments.

### 1.1. Series Arc Detection Techniques and Sensors

Several physical quantities, namely light, noise, current, temperature, and radiation, can be measured to detect the arc [20,21]; therefore, sensors, techniques, and methods have been explored related to every kind of emission. Some of them are summarised henceforth, including the type of sensor and commenting on its convenience for aeronautical use.

#### 1.1.1. Optical Methods—Light Monitoring

This method consists on capturing the flash emitted by the arc with photosensitive sensors, especially in the ultraviolet band, to obtain an electrical output that could trigger the control to open the endangered circuit [22,23]. The main disadvantage of optical methods is the need to measure close to the arc and, as a consequence, the great amount of devices required for cover the network onboard, making this solution hard to implement in aircraft. With the aim of mitigating this problem, fiber optic wires along the main conductors can be deployed [24,25], but still the installation issues remain a drawback.

#### 1.1.2. Acoustical Methods—Noise Monitoring

These are based on the use of microphones, piezoelectric sensors, or ultrasonic sensors, and are able to detect the sound waves produced by the arc, typically with a bandwidth up to 300 kHz. The discharge sound is used for triggering a protection mechanism [26], but again the need to be close to the fault and also the external acoustic perturbances make the implementation in aircraft quite complex.

#### 1.1.3. Electrical Methods—Impedance Monitoring

With reflectometry techniques, a high-frequency signal is sent down to the wire, where it reflects on any impedance discontinuity. The reflection coefficient gives a measure of how much signal is returned and gives the impedance of the discontinuity, where the arc (or other fault) has modified the wire [27]. Regarding aviation, the accuracy in the detection of the arc can be compromised by changes in the power circuit when new loads or joints are added to the original mesh. The signal reflection can also distort the waveform and make difficult the diagnosis. Furthermore, it is needed to invade the circuit with the test signal, what may entail several issues for operation and installation. In spite of these drawbacks, some ground test equipment has been developed based on this technique, which is useful for pre-flight wiring diagnosis, but does not address the real-time detection during aircraft operation.

#### 1.1.4. Electrical Methods—Current Monitoring through Magnetic Field Sensors

These techniques evaluate the currents through power cables and detect a variation when the arc occurs [28]. The arc affects the current signal and, as a consequence, provokes variations in its associated magnetic field, which is used to detect the arc. In AC and DC, this variation of the magnetic field can be studied in the range from kHz to MHz, with different kinds of sensors, including Hall effect sensors [29,30], antennas [31], and inductive sensors.

Inductive sensors measure the changes in the magnetic field using a coupling inductance. Traditionally, they have been used for low-frequency (LF) signals, from the range of kHz and even lower as in [32], where a current transformer is employed for series arc fault detection in residential buildings. In spite of that, these sensors can work in the high-frequency band (HF) (3 to 30 MHz) or in the very high-frequency band (VHF) (30 to 300 MHz). Different topologies of coils, including Rogowski coils [33], and HFCT (High Frequency Current Transformer) [34,35] are suitable for this application.

The magnetic field sensors offer several advantages to perform this task: they can provide good sensitivity, the monitoring of the cable is carried out with a non-invasive method based on passive sensors, the detection is more independent from the arc location, there are multiple size and weight options for sensors, the frequency detection range is wide, they can have fast responses, and it is possible to find options with no saturation. However, some issues can also be found, like false trips because of electromagnetic noise, Hall effect sensors being incapable of measuring currents above hundred kHz, or Rogowski coils having low sensitivity.

To perfect the arc detection solutions based on inductive devices, they typically integrate algorithms to analyse the output of the sensor [29,36,37], but, in any case, a good sensitivity is crucial for the overall performance of the solution and improves the implemented algorithm.

### 1.2. Proposed Approach and Background

Some of the previously labelled techniques have obtained promising results under a controlled laboratory environment; however, practical solutions impose additional challenges. Most of the designs have been elaborated for the implementation in ground vehicles, buildings, or photovoltaic modules, not considering the very important constraints for aeronautics, such as weight, size, environmental conditions, electromagnetic compatibility, safety, and reliability. As a consequence, attempting to locate the series arc fault during operation in HVDC networks onboard is a not satisfactorily solved issue yet.

Despite the difficulties, some good candidates have been identified for series arc fault detection in aviation. Detecting the changes in transported currents through power cables is considered one of the most suitable solutions for practical applications [28], and inductive sensors are the best candidates.

Inductive sensor solutions rely on Ampère’s and Faraday’s laws, which postulate that it is possible to have an electromotive force in the sensor dependent on the rate of change with time of the current through the main conductor, because of the magnetic flux variation. Thus, the effectiveness of the solution depends on the sensitivity of the sensor to those changes in the magnetic flux. Likewise, this sensitivity is related to the characteristics of the sensor namely materials, geometry, and installation. An optimal balance of these characteristics must be considered in the design to obtain the best configuration, because the better the sensitivity, the better the arc fault detection solution will be [38].

Traditionally, the studies for series arc fault detection in DC systems based on inductive sensors have been focused on the evaluation of frequencies up to 1 MHz. However, it is important to consider that the current pulses created by the high-voltage derivative in the inception of the arc and the quenching attempts along the duration of the arc are propagated along the transmission line of the electrical system. These HF current pulses inherit the frequency characteristics of the transmission line and can reach frequencies in the range of several tens of MHz, as demonstrated in [21,28,38]. Furthermore, in [38], the authors characterised the pulses in frequency, explained the significance of the length of wires and changes in load in the frequency components of the arc, and designed a light-weight sensor for the detection of HF signals from electric arcs propagated through the transmission line in 270 VDC systems, easily to install without galvanic contact to the power lines. Moreover, this band in the low range of MHz is relatively free of other interference in aeronautical systems. According to [39], there is a band between 2 MHz and 30 MHz where the highest levels of the allowed conducted RF interference are very low and this eases the monitoring and detection of the propagated pulses created by arcing.

Following the principles described in [38], this new study develops the concept with more precise equations and theoretical analysis, exploring different geometries for the sensor and elaborating representative modelling techniques for the design of arc fault detection solutions based on inductive sensors. With this aim, the next sections collect the calculations, modelling, and laboratory testing verification of the parameters influencing in the design of an inductive sensor intended for a series arc fault detector in aeronautical HVDC networks.

## 2. Inductive Sensor Modelling

### 2.1. Overview

**Figure 1:**
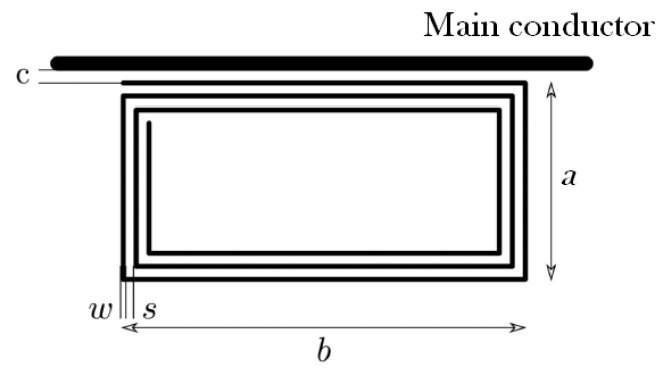
Three turns rectangular coil employed in [38] where *a* is the coil short side, *b* is the coil long side, *c* is the separation coil–main conductor, *s* is the separation between turns and *w* is the coil wire width.

The inductive sensor presented in [38] was designed with a series of four rectangular coils with three turns each; in Figure 1, one of the coils is represented. The sensor was printed on top and bottom faces of a circuit board that was placed along the main wire at both sides.

**Figure 2:**
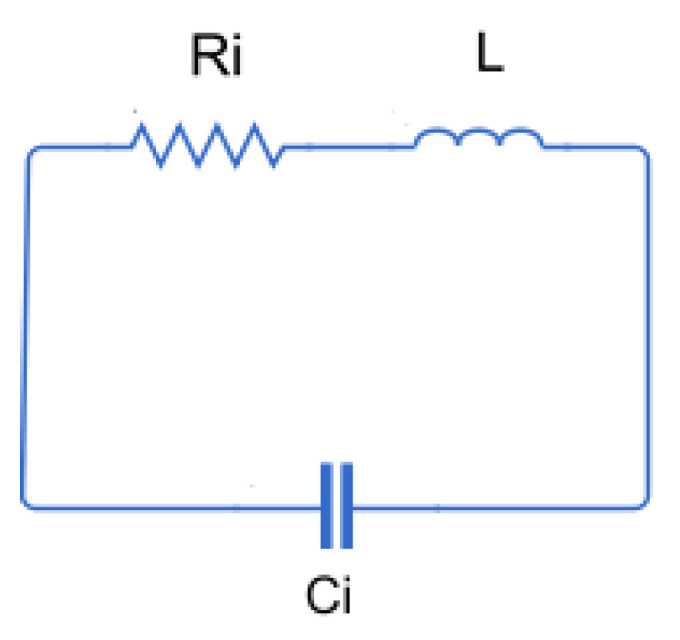
Equivalent circuit of a rectangular coil.

The equivalent circuit of a rectangular coil can be observed in Figure 2, with *L* being the self-inductance of the coil, $R_{i}$ its internal resistance, and $C_{i}$ the stray capacitance created by the wires of the parallel sides of the coil.

It is convenient to assess the relevance of $C_{i}$ to determine the effect of the resonance of the sensor on the frequency response. This capacitance can be calculated considering the stray capacitance of the long sides of the rectangular coil, $C_{b}$, and the stray capacitance of the short sides of the rectangular coil, $C_{a}$, in parallel. As an example, $C_{b}$ can be obtained with Equation (1):

(1) $$C_{b}=\frac{\pi \epsilon_{r}b}{\mathrm{arcCosh}\frac{a}{2r}},$$

where the relative permittivity $\epsilon_{r}$ can be considered is equal to 3, correspondent to the polylactic acid (PLA) 3D printed frame. And, for the coil of interest a=10 × $10^{-3}$ m, $b=90\times 10^{-3}$ m, and $r=0.1\times 10^{-3}$ m, so according to Equation (1), $C_{b}=1.63$ pF. Analogously, the stray capacitance of the short sides of the rectangular coil is $C_{a}=0.12$ pF. Thus, the resulting $C_{i}=C_{a}+C_{b}=1.75$ pF.

To complete the analysis of the stray capacitance influence in the inductive sensors of interest, it shall be taken into account that is foreseen to propose alternatives with several turns in the rectangular coil, disposed as parallel rectangles, which affects the total capacitance of the sensor. The rectangular turns will be separated at a distance d=2r+2δ, where δ is the coil wire insulation layer. Assuming $\delta =0.01\times 10^{-3}$ m and the permittivity of the copper wires is similar to the permittivity of the insulation, the stray capacitance of the two parallel rectangular coils $C_{p}$ is

(2) $$C_{p}=\frac{\pi \epsilon_{r}\left(2b+2b\right)}{\mathrm{arcCosh}\frac{d}{2r}},$$

yielding $C_{p}=12.54$ pF.

**Figure 3:**
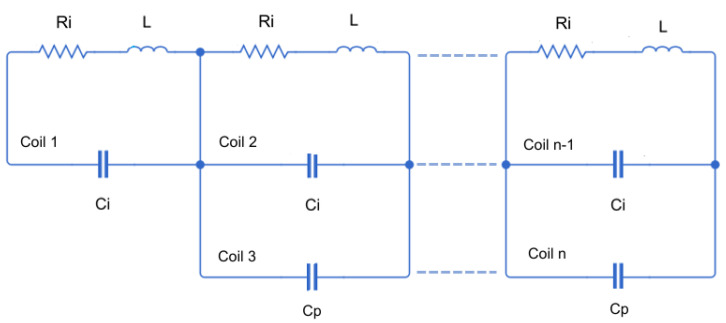
Equivalent circuit of *n* rectangular coils with several turns connected in series.

The last step consists of obtaining the total capacitance *C* of a sensor with *n* turns, which can be obtained from the solution of the equivalent circuit shown in Figure 3, and is expressed as follows:

(3) $$C=\frac{1}{\frac{1}{C_{i}}+\frac{n-1}{C_{i}+C_{p}}}.$$

From Equation (3), *C* can be quantified for some examples of coils disposed in series with different turns each, which for the capacitance calculation means a total of *n* rectangular coils (see Figure 3), resulting C=1.56 pF for n=2, C=1.28 pF for n=4, or C=1.09 pF for n=6. It can observed that, as the number of turns increases, the value of *C* is reduced and, in any case, *C* will be lower than $C_{i}$ because $C_{i}$ is in series with the rest of the capacitances, rendering the value of $C_{p}$ irrelevant. Therefore, it is concluded that in the most unfavourable case that would give the lowest self-resonance corresponds to a capacitance of $C=C_{i}=1.75$ pF. At the end of Section 2.2, it will be shown that the contribution of this capacitance does not interfere with the frequency response of the proposed sensors in the bandwidth of interest; consequently, the stray capacitance can be omitted in the equivalent circuit of the inductive sensor used to the rest of the analysis.

**Figure 4:**
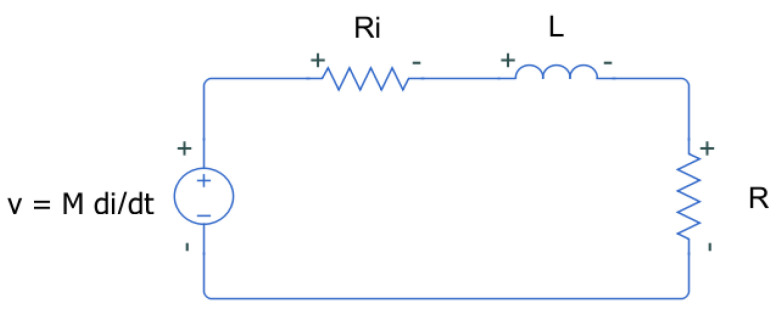
Equivalent circuit of a sensor induced by and external current *i* connected to an external load *R*.

The resulting equivalent circuit of the sensor, once induced by the main conductor current, corresponds to the diagram of Figure 4. The value of the gain *G* of the sensor is computed from the transfer function of this circuit.

The induced voltage is a function of the mutual inductance *M* between the main conductor and the sensor and the derivative of the current through the main conductor. The load *R* represents the input resistance of the acquisition system, whereas $R_{i}$ is the internal resistance of the coil.

Due to skin effect, $R_{i}$ depends on the frequency. In [40], the authors quantified the value of this resistance for a single rectangular loop, ranging it from 0.2 Ω to 0.8 Ω between 1 and 100 MHz.

Then, the transfer function between the current through the main conductor *I* and the output voltage $V_{0}$ of the sensor connected to *R* is

(4) $$G\left(s\right)=\frac{V_{o}\left(s\right)}{I(s)}=\frac{MsR}{Z_{s}+R}\approx \frac{Ms}{\frac{L}{R}s+1},$$

where $Z_{s}=Ls+R_{i}$, being the sensor impedance. Note that the transfer function can be simplified by considering that $R_{i}$ has a much smaller value than *R*.

This first order response has a pole in R/L that will define the bandwidth of the sensor. The bandwidth should fit in the frequencies of interest associated to the characteristics of the pulse and the transmission line, in this case is in a band from 100 kHz to 10 MHz, according to the range explored in [38]. This approach helps to suppress signals that can be in the DC system but are unrelated to the fault being detected [33], like conducted electromagnetic emissions allowed by the aeronautical standards [39].

The maximum gain *G* of the sensor can be obtained for sufficiently high frequencies as in Equation (4).

(5) $$G=\frac{MR}{L}.$$

From this expression, it can be inferred that *L* and *M* are essential parameters for the voltage output of the sensor. As a consequence, seeking a convenient value of *L* and *M* is essential for the design of the inductive sensor.

Considering that the media for the magnetic field created by the current pulses is air, so it is linear, the self-inductance *L* equals the ratio of the magnetic flux linkage through the *N* turns coil ϕ to the current in the main conductor *i* [41]:

(6) $$L=N\frac{\phi }{i}.$$

On the other hand, according to [42,43], the mutual inductance *M* between a rectangular coil and an infinite long main conductor can be obtained from

(7) $$M=N\frac{\mu_{0}}{2\pi }b\ln\frac{a+c}{c},$$

where $\mu_{o}$ is the magnetic permeability of free space, *a* is the coil short side length, *b* is the coil long side length, and *c* the separation distance between coil and main conductor.

The results obtained by the authors in [38] evidenced that the use of several rectangular coils offered values for total *L* and *M* pertinent for the detection of the HF pulses created by the arc.

Under this premise, the first objective of this study is to obtain a representative model for *L* mathematically and using the Finite Elements Method (FEM). Secondly, following the same techniques, the validation of *M* models is performed. The results will be useful to develop improved geometries for the arc fault detection sensors in terms of sensitivity, weight and size.

**Figure 5:**
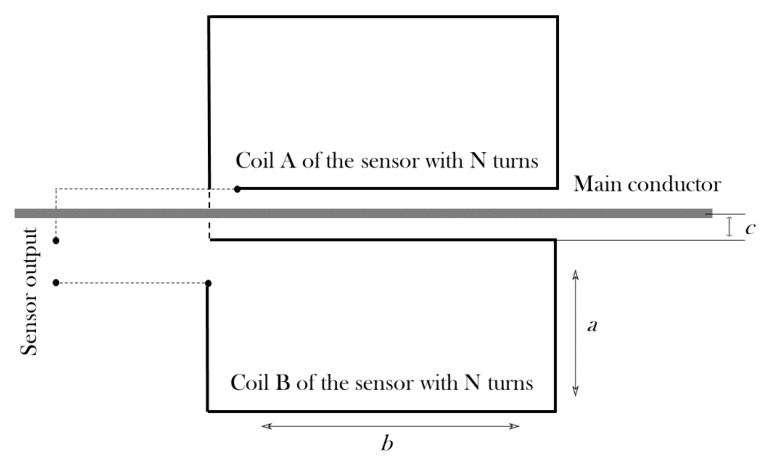
Proposed geometry for an inductive sensor with two rectangular coils with *N* turns each, where: *a* is the coil short side, *b* is the coil long side, and *c* is the separation coil–main conductor.

The geometries evaluated in this paper are based on two rectangular coils in series of *N* turns each placed at both sides of the main conductor. As mentioned before, The proposed dimensions for the rectangular coil are: a=10 × $10^{-3}$ m, $b=90\times 10^{-3}$ m and, now $d=0.2\times 10^{-3}$ m, with *d* the wire diameter neglecting the enamel. The study will be carried out with three sensors based in rectangular coils: sensor#1, with two coils of one turn each (N=1), sensor#2, with two coils of two turns (N=2) and sensor#3, with two coils of three turns (N=3), separated $c=3.5\times 10^{-3}$ m from the axis of the main conductor, see Figure 5. The separation *d* between the turns in the coils with N=2, and N=3 is the diameter of the wire. The aim is to understand how the number of turns changes the frequency response of the sensors and to find what number is the most suitable to detect the current pulses derived from the arc with the highest sensitivity.

The first step to evaluate the performance of the sensors is to carry out a theoretical approach, which will be useful to consolidate the subsequent FEM analysis. A second step will consist of the construction and execution of the representative FEM model, comparing results with the theoretical approach. Finally, a real prototype will be tested in the laboratory, and the model predictions will be validated.

### 2.2. Theoretical Approach

#### 2.2.1. Inductance of a Single Rectangular Coil

Since the different proposed sensors are based on rectangular coils, it shall be firstly computed the self-inductance of a single rectangle, and then to obtain the total self-inductance for the three sensors under study. The equation that gives the value of the self-inductance of a rectangular loop is based on the works of Rosa and Grover [42,43,44]. The basis is simply the summation of the self-inductances of individual straight thin cables and the mutual inductances between them. The self-inductance of a straight copper wire with circular section with radius *r*, $L_{s}$, is given by

(8) $$L_{s}=0.2b\ln\frac{2b}{r}-k+\frac{r}{b}.$$

The constant *k* depends on the frequency and the shape of the conductor ranging from 0.75 for low frequencies or DC signals to 1 for high frequencies, where the magnetic field within the conductor is reduced [42]. In this study, *k* has been considered equal to 1, which is a valid value for round wires and the frequencies of interest already mentioned.

The self-inductance of a single copper rectangle loop, $L_{r}$, with round wires of breadth *a* and length *b*, comes from the summation of self-inductances of the parallel straight wires, and the correspondent mutual inductances of the opposite pairs of length *a* and *b*, being $L_{r}=L_{a}+L_{b}-M_{a}-M_{b}$ [42]. The development of this formula, according to [42], yields

(9) $$\begin{array}{cc} L_{r}= & 0.4\left.[\left(a+b\right)\ln\frac{2ab}{r}-a\ln\left(a+g\right)\right.\\ \; & \left.-b\ln(b+g)-2(a+b)+2(g+r)\right.], \end{array}$$

Being $g=\sqrt{a^{2}+b^{2}}$. With the distances expressed in meters, the obtained self-inductance magnitude is μH.

Then, with the dimensions of the selected geometry for the study (as described in Figure 5), the resulting self-inductance of the single rectangular coil is $L_{1}=178.9$ nH.

#### 2.2.2. Sensor #1 Theoretical Self-Inductance

The sensor#1 geometry is made up of two one-turn rectangular coils connected in series, with a total self-inductance $L_{11}=2L_{1}+M_{11}$, where $L_{1}$ is the self-inductance of each one of the coils obtained in the previous section in Equation (9), and $M_{11}$ is the mutual inductance between the two rectangular coils. This parameter $M_{11}$ is the summation of the partial mutual inductances between the parallel wires of the two rectangles, [42], which is repeated here for the sake of clarity

(10) $$M_{p}=0.2\left(l\ln\frac{l+h}{s}-h+s\right),$$

where *l* is the length of the side of the rectangle under consideration, *s* is the distance that separates them which can be *a*, a+2c and 2a+2c and $h=\sqrt{l^{2}+s^{2}}$. $M_{p}$ is calculated for each side of the rectangular coil.

**Figure 6:**
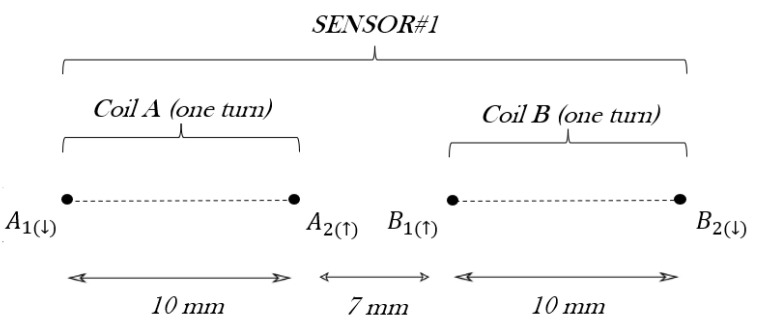
Schematic of sensor#1 including a cross-section view of the coils.

Figure 6 shows the cross-section of the sensor, highlighting the wires used for the calculation of $M_{11}$: $A_{1}$, $A_{2}$, $B_{1}$, and $B_{2}$. Only the parallel long sides of the rectangles have been considered, because the mutual inductances between the short sides are negligible. The direction of the current flowing through the coils has been also represented as (↑) or (↓), given that it is necessary to identify if the partial mutual inductance between two wires shall be added (same direction) or subtracted (opposite direction).

Consequently, $M_{11}=M_{A_{1}B_{2}}-M_{A_{1}B_{1}}+M_{A_{2}B_{1}}-M_{A_{2}B_{2}}$, what yields 15.06 nH. As a consequence, the sensor#1 theoretical self-inductance is given by: $L_{11}=2L_{1}+M_{11}=2\times 178.9+15.06=372.9$ nH.

#### 2.2.3. Sensor#2 Theoretical Self-Inductance

The sensor#2 geometry is made up of two rectangular coils, with two turns each. Following the same principle explained for sensor#1, the total self-inductance of sensor#2 $L_{22}$, is given by $L_{22}$ = $2L_{2}+M_{22}$, where $L_{2}$ is the self-inductance of a rectangular coil with two turns and $M_{22}$ is the mutual inductance between the rectangles at both sides of the main conductor.

**Figure 7:**
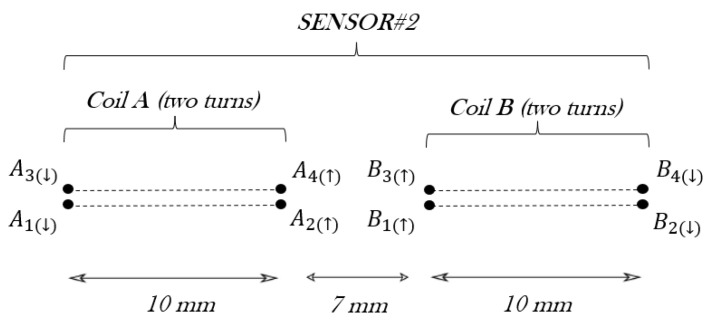
Schematic of sensor#2 with a cross-section of the coils.

$L_{2}$, considering only coil A in Figure 7, is obtained through the self-inductance of two rectangles in series $2L_{1}$, which was already calculated for sensor#1, and the mutual inductance between two rectangular turns one on the other $M_{2}$, found in [42] and repeated in Equation (11):

(11) $$\begin{array}{cc} M_{2}= & 0.4a\ln\left(\frac{a+\sqrt{a^{2}+d^{2}}}{a+\sqrt{a^{2}+b^{2}+d^{2}}}\frac{\sqrt{b^{2}+d^{2}}}{d}\right)\\ \; & +0.4b\ln\left(\frac{b+\sqrt{b^{2}+d^{2}}}{b+\sqrt{a^{2}+b^{2}+d^{2}}}\frac{\sqrt{a^{2}+d^{2}}}{d}\right)\\ \; & +0.8\left(\sqrt{a^{2}+b^{2}+d^{2}}-\sqrt{a^{2}+d^{2}}-\sqrt{b^{2}+d^{2}}+d\right), \end{array}$$

where *d* is the diameter of the wire and the separation between the two rectangles.

Therefore, $L_{2}=2L_{1}+2M_{2}$. The values of these inductances for coil A are $M_{2}$ = 151.3 nH and $L_{2}=2\times 178.9+2\times 151.3=660.4$ nH.

The next step is to calculate the mutual inductances between coil A and coil B, $M_{22}$ using Equation (10). In this occasion, the parallel wires to obtain $M_{22}$ are represented in Figure 7, being $A_{1}$…$A_{4}$ and $B_{1}$…$B_{4}$. The combination of mutual inductances between these eight wires gives $M_{22}$ = 60.24 nH. Then, the total self-inductance of sensor#2 results are as follows: $L_{22}=2L_{2}+M_{22}=2\times 660.4+60.24=1381.04$ nH, since coils A and B are in series.

#### 2.2.4. Sensor#3 Theoretical Self-Inductance

The sensor#3 geometry is made up of two rectangular coils, of three turns each. Again, the total inductance of the sensor depends on the self-inductance of one rectangular coil, e.g., coil A, with three turns $L_{3}$ and the mutual inductance between coil A and coil B coils $M_{33}$, following the expression: $L_{33}$ = 2$L_{3}$ + $M_{33}$.

The self-inductance of a rectangular coil with three turns is $L_{3}=3L_{1}+M_{3}$, where $M_{3}$ is analogous to $M_{2}$ and can be found in [42]. Now, this mutual inductance is the sum of three partial mutual inductances between the three rectangular turns to yield $M_{3}=$ 852.72 nH. In consequence: $L_{3}=3\times 178.9+2\times 852.72=1389.42$ nH.

**Figure 8:**
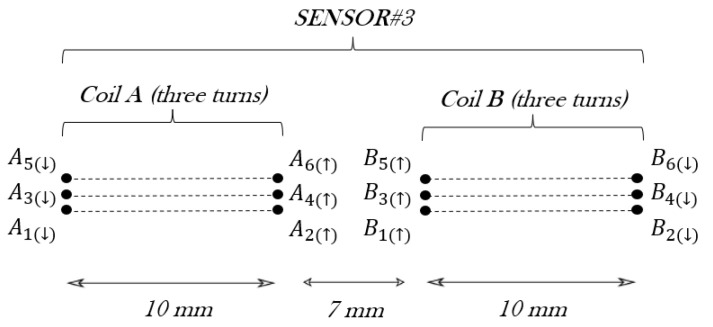
Schematic of sensor#3 with a cross-section of the coils.

Next, the mutual inductance between coils A and B $M_{33}$ considers the wires $A_{1}$…$A_{6}$ and $B_{1}$…$B_{6}$ represented in Figure 8. Following the sequence of the results for $M_{11}$ and $M_{22}$, the total mutual inductance between two rectangular coils of *N* turns can be written as $M_{NN}=N^{2}M_{11}$. Therefore, $M_{33}=3^{2}\times 15.06=135.54$ nH. So the total self-inductance of sensor#3 results are as follows: $L_{33}=2\times 1389.42+135.54=2914.38$ nH.

#### 2.2.5. Theoretical Mutual Inductance between Sensor and Main Conductor

Once the theoretical value of the self-inductance of sensor#1, sensor#2, and sensor#3 is obtained, the next step to complete the transfer function of the equivalent circuit is to know the mutual inductance between each sensor and the main conductor.

In this disposition, the calculation of the mutual inductance *M* can be derived considering the magnetic flux ϕ created by the current *i* passing through *N* loops, which is obtained from Equation (6). Then, the next expression gives *M* for N rectangular turns:

(12) $$M=N\frac{\mu_{0}}{2\pi }b\ln\frac{a+c}{c},$$

where $\mu_{0}$ is considered the permeability of copper, and $c=3.5\times 10^{-3}$ m (see Figure 5).

Accordingly, the mutual inductance for one coil with one loop can be obtained from Equation (12), yielding: $M_{01}=24.3$ nH. Similarly, according to Equation (7), for one coil with two loops $M_{02}=48.6$ nH and for one coil with three loops $M_{03}=72.5$ nH. Taking into account that the proposed sensors have two coils on both sides of the main conductor connected in series, the induced voltages are added. Thus, the total mutual inductance is obtained by multiplying by 2, resulting in the values expressed in Table 1.

#### 2.2.6. Theoretical Frequency Response of the Proposed Sensors

**Figure 9:**
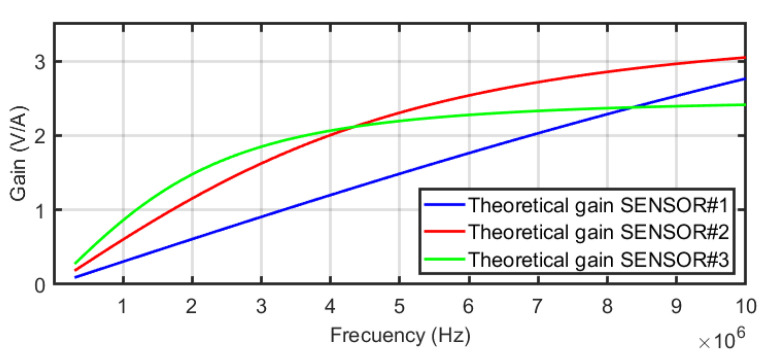
Frequency responses of the three sensors.

The frequency response of the equivalent circuit of the sensors in Figure 4 was defined in Equation (4). This equation is the transfer function between the output of the sensor connected to *R* to the current through the main conductor, assuming *R* = 50 Ω the input resistance of any high-frequency acquisition system. Figure 9 shows the frequency response from 100 kHz to 10 MHz for the three sensors using the self-inductances of the theoretical model. The comparative analysis offers very useful information about the performance of the different sensors when the number of turns is increased. For instance, the bandwidth is extended to lower frequencies for N=3 but the sensitivity at higher frequencies decreases as expected from Equation (5) since the self-inductance increases and the pole in R/L moves to the left. The plot for N=1 is a pure derivative in this range of frequencies and the sensor with N=2 has the best sensitivity for frequencies above 4.2 MHz. The modelling helps in the decision to select one specific sensor with the best characteristics in terms of bandwidth and sensitivity to detect arcs which is the final goal.

The influence of the stray capacitance self resonance in the frequency response of the sensor has been also evaluated. In the worst case scenario, considering the highest inductance, which is $L_{33}=2914.38$ nH, and considering the highest stray capacitance $C_{i}=1.75$ pF obtained in Section 2.1, the self resonance frequency $f_{r}$ has been calculated following the next expression:

(13) $$f_{r}=\frac{1}{2\pi \sqrt{L_{33}C_{i}}},$$

Giving $f_{r}=70.6$ MHz, which is quite far from the bandwidth of interest, confirming the omission of the stray capacitance in the analysis of the equivalent circuit of the sensors.

### 2.3. FEM Analysis

**Figure 10:**
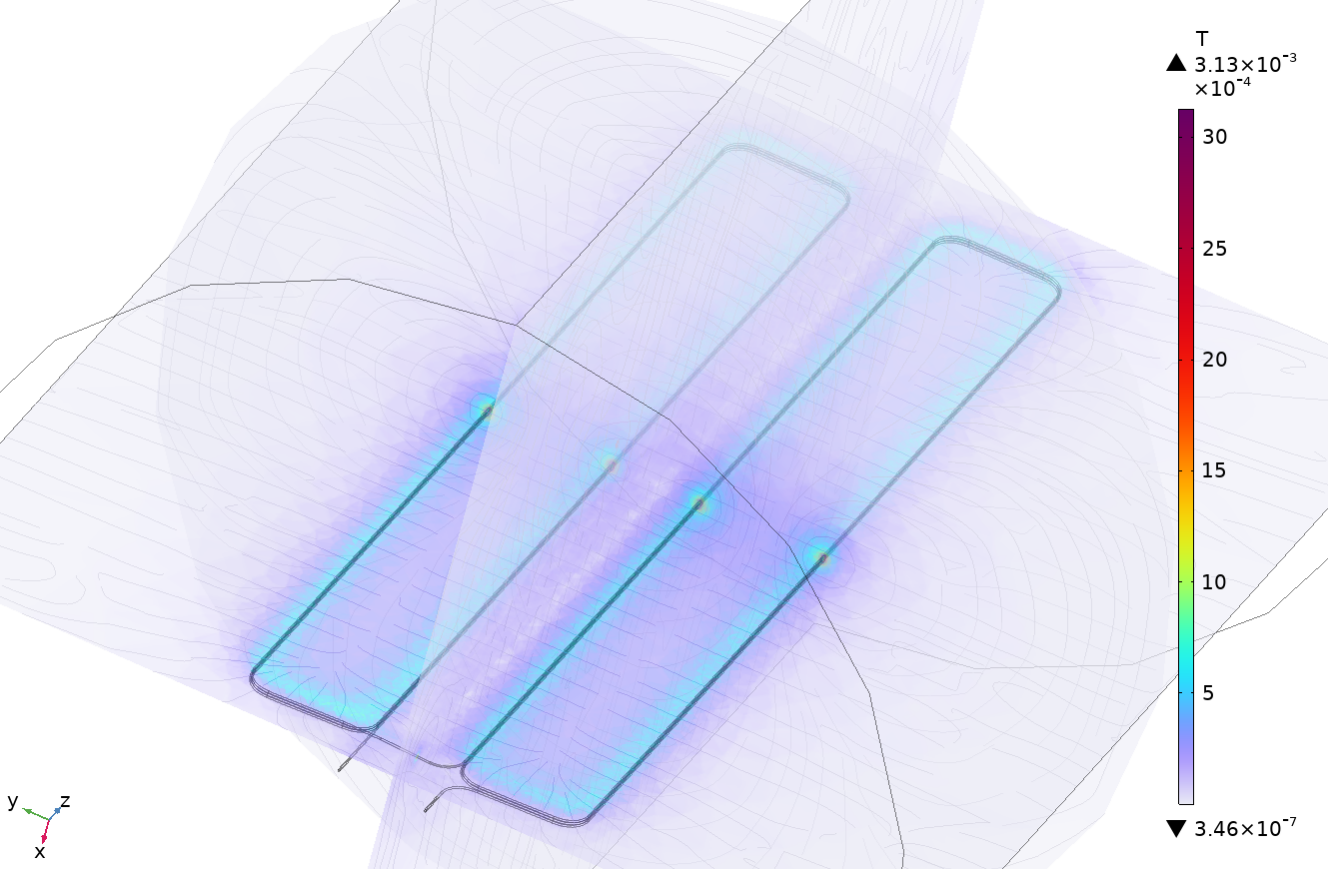
Magnetic flux density distribution in the 3D model of sensor#3 for a sinusoidal test current through the main conductor at 1 MHz.

Three FEM models have been developed for the evaluation of the sensors using a software for multiphysics simulation, Figure 10. The objective is to obtain their self-inductances to validate the theoretical model which is easier to implement in a future optimisation process of the geometry of the sensors. In the definition of the FEM models, the sensor is surrounded by a cylindrical volume of air, with a height of $100\times 10^{-3}$ m and a base with a radius of $40\times 10^{-3}$ m, large enough to be considered infinite in terms of magnetic effects. In addition, the boundaries have been considered as infinite elements, avoiding the reflection of the magnetic field.

**Figure 11:**
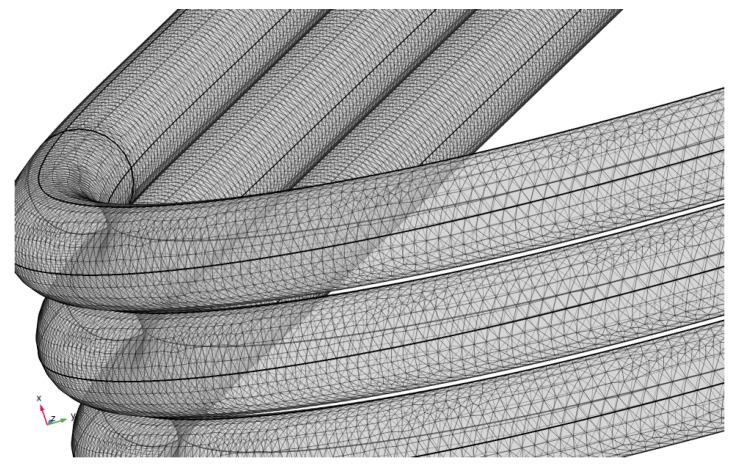
Detailed mesh of inductive sensor with coils of three turns (sensor#3).

The three virtual geometries have been evaluated using a coil geometry analysis that employs magnetic field equations, like Ampère’s law. The geometry of the sensors have been decomposed in an extra fine tetrahedral mesh, with a minimum element size of $0.1\times 10^{-3}$ m, as can observed in Figure 11, which is required for a high accuracy in the computation of the self-inductance.

The study for each geometry has been carried out in the frequency domain, circulating a probe base current at 1 MHz through the sensor. This frequency is consistent with the assumed value for *k* in Equation (8). It was also verified that there were no significant differences in the results of the model using k=1 with frequencies up to 10 MHz.

To obtain the self-inductance, the simulation uses the probe current through the coil and the coil concatenated magnetic flux coming from the magnetic flux distribution (see Figure 10). The resultant modelled self-inductance for each sensor is exposed in Table 2, where they are compared with the self-inductances obtained in the theoretical approach; the relative error between them is included. Taking into account the small difference, the simulation adherence to the analytical calculations from Section 2.2 is good enough to consider the model as representative.

## 3. Experimental Study of the Proposed Sensors

### 3.1. Transfer Function Experimental Measurement

Functional prototypes of the sensor have been manufactured with additive printing in 3D and wound for the experimental characterisation of their properties in frequency. Now, the aim is to corroborate both the theoretical and FEM models.

The frequency response was obtained with a vector network analyser (VNA) measuring the transimpedance, $Z_{TF}$, output voltage for a known input current through a 50Ω load, of the three probes. The frequency study spans from 300 kHz, which is the minimum frequency allowed by the VNA to 10 MHz. The complex values of the reflection coefficient at port 1 $S_{11}\left(f\right)$ and the transmission coefficient $S_{21}\left(f\right)$ were obtained to calculate the sensors transimpedance [45] with Equation (14).

(14) $$Z_{TF}=\frac{|S_{21}|\times 50}{|1-S_{11}|}.$$

**Figure 12:**
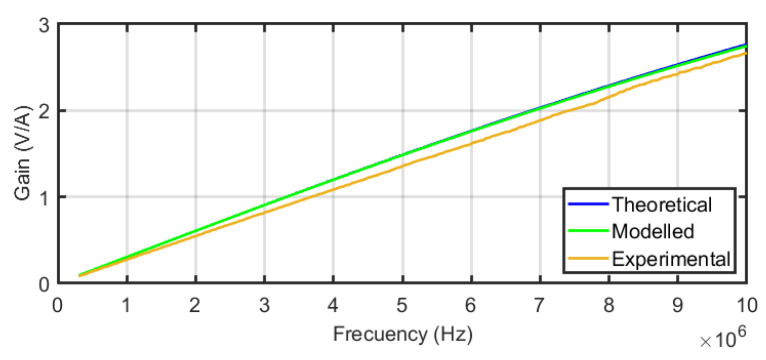
Compared frequency response of sensor#1 for the theoretical and FEM models and experimental results.

**Figure 13:**
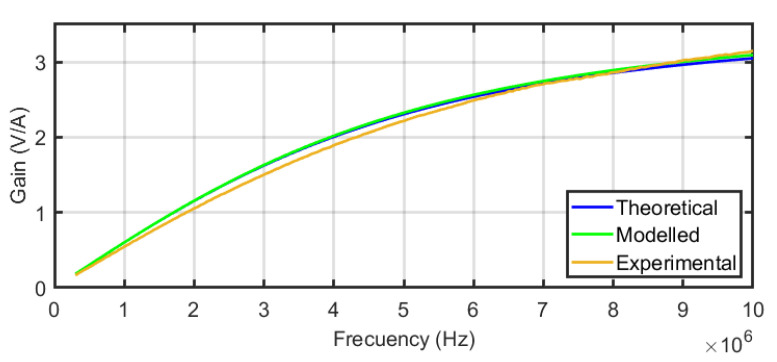
Compared frequency response of sensor#2 for the theoretical and FEM models and experimental results.

**Figure 14:**
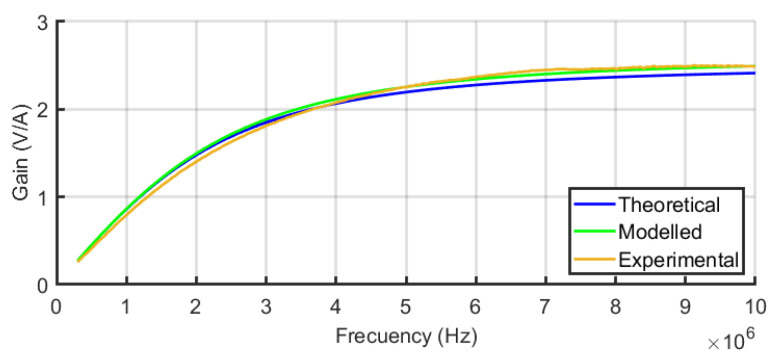
Compared frequency response of sensor#3 for the theoretical and FEM models and experimental results.

The resulting experimental transimpedances versus frequency for the sensors are compared with their theoretical and their FEM modelled frequency responses in Figure 12 for one turn, Figure 13 for two turns, and Figure 14 for three turns. In order to quantify the deviation of the experimental results with the theoretical and modelled responses, the mean squared error has been calculated for each figure. The square root of the mean squared errors has been obtained to represent the deviation in volts per ampere. The results are collected in Table 3, which reveals the good coincidence between the theoretical approach with the experimental measurements (upper row of the table) and between the FEM model with the experimental measurements (bottom row of the table), both for the three sensors.

Therefore, the models can be assumed as representative of the real performance. This opens a path to design a sensor with geometric and electrical parameters adaptable to the characteristics of the signal to measure which in our case is the propagation of the HF current pulses created by a serial arc through a cable.

### 3.2. Experimental Detection of Series Arcs with the Sensor Prototypes

**Figure 15:**
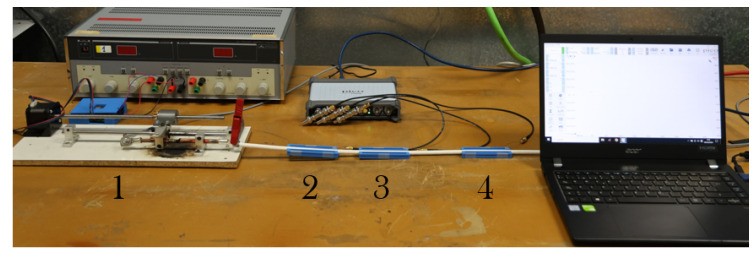
Experimental tests setup including: arc fault generator (1), sensor#1 (2), sensor#2 (3), and sensor#3 (4); the laptop that houses the acquisition software can also be observed.

To validate the functionality and the practical applicability of the current sensor design, all sensor prototypes have been tested with real series arcs created in the High-Voltage Laboratory at the University Carlos III of Madrid. For this purpose, a 2 kW variable resistive load was connected to a 270 VDC voltage source and adjusted to a 5 A current level. The sensors have been installed along a representative wire used in aircraft installations with a total gauge of $7\times 10^{-3}$ m including $1\times 10^{-3}$ m of insulation, that powers the load. The circuit can be interrupted with a remotely controlled arc fault generator, already described by the authors in a previous work [38], which can create series arcs in the power line. This setup can be observed in Figure 15.

**Figure 16:**
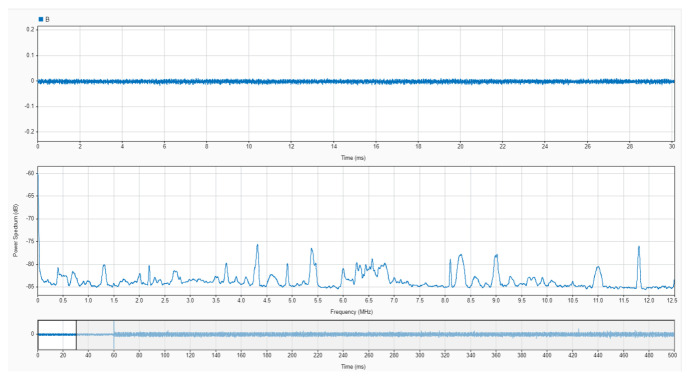
Capture of an experimental measurement with sensor#2 previous to arcing in the monitored circuit. (**Upper plot**) time-domain signal from the selected time range; (**middle plot**) average power spectrum from the selected time range; (**lower plot**) selected time domain range from the overall signal.

**Figure 17:**
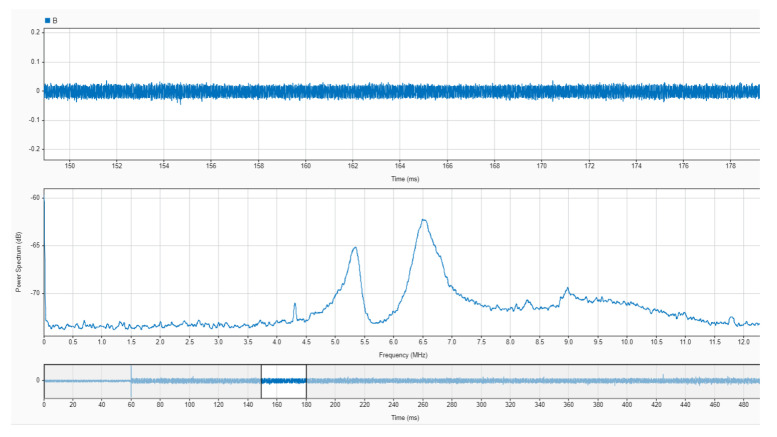
Capture of an experimental measurement with sensor#2 when arcing in the monitored circuit. (**Upper plot**) time-domain signal from the selected time range; (**middle plot**) average power spectrum from the selected time range; (**lower plot**) selected time domain range from the overall signal.

An oscilloscope with a resolution of 14 bits was used to acquire the output signal from the sensor with a sampling frequency of 25 MS/s in time windows of 500 ms. The acquisition was triggered with the sudden increase in a current pulse in the opening of the electrodes. The pre-trigger was set to 12% to obtain 60 milliseconds of no arc signal, as shown in Figure 16. In Figure 16 and Figure 17, the upper plot corresponds to the signal in the time domain corresponding to the selected interval shown in the bottom plot. The middle plot is the power spectral density in dB of the signal in the selected interval. It is precisely in this plot where the information needed to characterise the monitored signals as arc or no arc is found. A good measurement to detect an arc consists on comparing the power spectral density of the HF current signals when there is no arc and when there is an arc. An example of this comparison has been conducted for sensor#2 in Figure 16 and Figure 17. It can be seen an important rise in the spectral power for all frequencies with a noticeable growth in the base level of spectral power when arcing compared with no-arc. There is also an increment of power from 5 MHz to 10 MHz, especially with the appearance of two resonances around 5.3 MHz and 6.5 MHz. As shown in [38], peaks at certain frequencies are related to the experimental setup: cable lengths, cross sections, and, especially, the number and location of joints. According to the results for the frequency response of the sensors shown in Figure 12, Figure 13 and Figure 14, it can be inferred that the sensor#2 is the best candidate for this setup because its sensitivity at those frequencies is the highest of the three. In other layouts, especially those with larger cables, the frequency content of the HF current components can be shifted to lower frequencies, so the sensor#3 would be a better candidate. This dependence of the circuit characteristics and elements on the frequency characteristics of the signals, anticipates the need for the customisation of the sensors for arc detection to obtain the best sensitivity and bandwidth. This confirms the relevance of having representative models that help in the design of the sensors.

Five measurements of arcs have been taken for each sensor to conduct a reliable quantification of the phenomenon. The power of each signal was calculated in a frequency band spanning from 100 kHz to 10 MHz before and after the inception of the arc and then averaged. Table 4 contains a summary of the power differences between the signals with arc and without arc. The first column is the power of the first 60 milliseconds of the signal which corresponds to the pre-trigger without arc. The second column is the power in the next 60 milliseconds just after the trigger and the third column is the ratio between both powers. The results corroborate that both sensors with two and three turns are good candidates to measure arcs in the setup under test, with sensor#2 being the most appropriate as already demonstrated according to the frequency content of the arc signal.

## 4. Conclusions

The suitability of inductive sensors for series arc fault detection in DC networks in aviation has been studied and it is considered one of the most promising sensors to address this topic. Since the sensitivity and bandwidth of the inductive sensors are key for the detector performance and the geometry of the sensor is directly related to the sensitivity and bandwidth, to find efficient and reliable techniques for the design of different geometries alternatives becomes crucial to develop practical applications. The variations in the geometry will entail variations in the self-inductance, which will also determine the frequency response of the sensor. This paper was focused on a theoretical and an FEM model for the design of air-core inductive sensors with required frequency response for arc detection. The modelling of the geometry and number of turns with FEM corroborates the results for the self-inductances obtained with the theoretical model. Additionally, three sensors using additive printing in 3D were manufactured and characterised in the laboratory measuring their frequency responses. The small deviation between models and experimental calculations shown in Table 3 demonstrates a very good agreement validating the accuracy of the modelling method. Finally, the performance of the sensors under real arcs in a setup with a portion of cable used in aircraft was tested, demonstrating that the sensors can detect the HF current pulses derived from the arc. It was also found that sensor#2 and sensor#3 were the most appropriate candidates to detect the arc, being sensor#2 the best option for that particular setup. Once the models are validated, the next step will be to find the best dimensions of the sensor to enhance its sensitivity and bandwidth in the detection of arcs. The information obtained in ongoing work is crucial for the design and selection of arc fault detection devices in more-electric and all-electric aircraft.

## Figures and Tables

**Table 1:** Mutual inductance of each sensor with the main conductor.

	Sensor#1	Sensor#2	Sensor#3
Theoretical *M*	$M_{1}=48.6$ nH	$M_{2}=97.2$ nH	$M_{3}=145$ nH

**Table 2:** Self-inductance for each sensor obtained from the models.

	Sensor#1	Sensor#2	Sensor#3
FEM Modelled	$L_{11}=386$ nH	$L_{22}=1355$ nH	$L_{33}=2814$ nH
Theoretical	$L_{11}=373$ nH	$L_{22}=1381$ nH	$L_{33}=2914$ nH
Error	3.37%	1.92%	3.56%

**Table 3:** Square root of the mean squared error for each sensor, indicating, in volts per ampere, the deviation of the experimental results with the theoretical and modelled.

	Sensor#1	Sensor#2	Sensor#3
Experimental–theoretical deviation (V/A)	0.0078	0.0055	0.0056
Experimental–modelled deviation (V/A)	0.0074	0.0059	0.0035

**Table 4:** Power increase in the signals from no arc to arc to evaluate the sensor performance in terms of bandwidth and sensitivity.

	No-Arc		Arc		Ratio
	$V^{2}\times 10^{-6}$	dB	$V^{2}\times 10^{-6}$	dB	dB
Sensor#1	5.0	−53	53.2	−42.7	10.2
Sensor#2	2.9	−55.4	53.4	−42.7	12.7
Sensor#3	4.2	−53.8	66.5	−41.8	12

## Data Availability

Data are contained within the article.

## Abbreviations

The following abbreviations are used in this manuscript:

AC	Alternating Current
AEA	All-Electric Aircraft
AFCB	Arc Fault Circuit Breaker
DC	Direct Current
FEM	Finite Elements Methods
HF	High Frequency
HFCT	High-Frequency Current Transformer
HVDC	High-Voltage Direct Current
MDPI	Multidisciplinary Digital Publishing Institute
MEA	More-Electric Aircraft
PLA	Polylactic Acid
VDC	Voltage Direct Current
VHF	Very High-Frequency
